# Enhancing NAD^+^ Metabolome in Cardiovascular Diseases: Promises and Considerations

**DOI:** 10.3389/fcvm.2021.716989

**Published:** 2021-08-27

**Authors:** Fahmida Jahan, Rushita A. Bagchi

**Affiliations:** ^1^Department of Biochemistry, Microbiology and Immunology, University of Ottawa, Ottawa, ON, Canada; ^2^Division of Cardiology, Department of Medicine, University of Colorado Anschutz Medical Campus, Aurora, CO, United States

**Keywords:** nicotinamide adenine dinucleotide, metabolome, cardiovascular disease, aging, therapeutic

## Introduction

Nicotinamide adenine dinucleotide (NAD) is not only the master regulator of energy metabolism pathways but also an essential substrate for several key enzymes of health and disease states (1, 2). NAD^+^/NADH redox coupling is vital for fueling glycolysis, tricarboxylic acid (TCA) cycle and electron transport chain (ETC) for producing ATP. In addition to that, NAD^+^ is a substrate for NAD-dependent deacetylases called sirtuins (SIRTs), poly-ADP-ribose polymerases (PARPs), monoADP-ribosyltransferases (ARTDs), ADP ribose synthetases (CD38/CD157), and sterile alpha and toll/interleukin-1 receptor motif-containing protein 1 (SARM1), etc. (1). NAD kinase also utilizes NAD^+^ to generate nicotinamide adenine dinucleotide phosphate (NADP^+^/NADPH) which is consumed by reactive oxygen species producing enzymes, NADPH oxidases. For maintaining cellular homeostasis, NAD^+^ levels are tightly regulated through its salvage pathway where the by-product of enzymatic reactions, nicotinamide adenine dinucleotide (NAM), is converted back to NAD^+^ by the rate limiting enzyme NAM phosphoribosyl transferase (NAMPT) (1, 2). Other biosynthesis pathways include *de novo* synthesis from tryptophan (Trp), and from the dietary uptake of its precursors- nicotinic acid (NA) or nicotinamide riboside (NR) (1, 2).

## NAD^+^ Depletion in Aging and Disease States

Maintaining the levels of NAD^+^ is highly critical for normal cellular function and this is indicated by the embryonic lethality of homozygous knockout of the NAD^+^ salvage enzyme *Nampt* in mice (3). Various tissue-specific conditional knockout *Nampt* mice also demonstrated the importance of maintaining NAD^+^ levels within an organ (Table 1). During metabolic stress, chronic inflammatory conditions, and aging NAD^+^ content has been shown to decline (1, 10, 11). In healthy humans, plasma NAD^+^ levels and the levels of its related metabolites such as NADP^+^ and nicotinic acid adenine dinucleotide (NAAD) have been reported to decline with age (12). Several studies have shown that with aging, the major NAD^+^ consuming enzymes such as PARP1 and CD38 are hyperactivated due to age-related increase in DNA damage, inflammation and oxidative stress leading to impaired NAD^+^ signaling (1, 11, 13, 14). In mouse models of muscular dystrophy, increasing levels of NAD^+^ was shown to improve muscle function, decrease muscle stem cell senescence, and increase lifespan (15, 16). In aging- related neurodegenerative diseases replenishment of NAD^+^ improved health span by reducing neuroinflammation and DNA damage in mouse brain (17–19). In an animal model of high-fat high-sucrose (HFHS) diet, NAD^+^ supplementation prevented hepatic steatosis by activating sirtuin mediated mitochondrial unfolded protein response (UPRmt) (20). NAD^+^ boosting was also shown to be beneficial for type 2 diabetes and diabetic neuropathy in mice (21). A clinical trial showed that NR can improve physical activity acutely (22) and chronic NR supplementation has been shown to decrease levels of inflammatory cytokines in sera of aged humans. NR also enhanced the levels of NAD^+^ and its related metabolites in muscle (10). In contrast, some clinical trials did not observe beneficial effects of NAD^+^ boosting in certain disease conditions. For instance, in obese men, either non-diabetic or insulin resistant, NR supplementation for a period of 12 weeks did not improve insulin sensitivity or glucose tolerance (23, 24). Moreover, the research group did not find any changes in skeletal muscle mitochondrial function or content in these subjects (25). This suggests that the beneficial effect of NAD^+^ supplementation may be tissue-specific or may require a long-term and dosage-optimized treatment regime, or may be in certain conditions it could be only effective as a preventative measure rather than a therapeutic (26).

**Table 1:** Organ specific knockout models of NAD+ salvage enzyme *Nampt* and their phenotypes.

**Organ/cell type**	**Phenotype**	**References**
Adipose tissue	No change in body composition but reduced food intake in high fat diet fed mice and improves glucose tolerance	(4)
Hepatocyte	50% reduction in total liver NAD^+^ levels and 20% reduction in mitochondrial NAD^+^ levels in hepatocytes; had minimal effect on liver mitochondrial function as *de novo* NAD^+^ synthesis is increased in the mitochondria as a compensatory mechanism	(5)
Renal proximal tubule	Tubular fibrosis characterized by tubular basement membrane (TBM) thickening and collagen deposition	(6)
Adult projection neurons	Weight loss, motor dysfunction, and death	(7)
Hypothalamic Agouti-related protein (AgRP) neuron	Reduced ATP levels, increased oxidative stress, and cell death leading to neuronal degeneration	(8)
Skeletal muscle	Mice were smaller in size with muscular dystrophy-like phenotype causing premature death. Impaired Ca^2+^ signaling and mitochondrial dysfunction were observed	(9)

## NAD^+^ Boosting in Cardioprotection

Ischemia, ischemia reperfusion (IR) injury, or myocardial infarction has been shown to result in reduced NAD^+^ levels in the heart (27). Friedreich's ataxia cardiomyopathy mouse model has been shown to be associated with increased mitochondrial protein acetylation along with a decrease in mitochondrial Sirt3 deacetylase transcript levels as well as altered expression of NAD^+^ biosynthesis enzymes. The authors demonstrated that NAD^+^ boosting by NR normalized cardiac efficiency by activating mitochondrial Sirt3 function and thereby reducing mitochondrial protein acetylation (28). Moreover, a mouse cardiac hypertrophy model showed that NAD^+^ treatment can inhibit pro-hypertrophic pathway and activate anti-hypertrophic LKB1-AMPK pathway by Sirt3-dependent deacetylation of LKB1 (29). Mouse models of dilated cardiomyopathy and cardiac hypertrophy showed 30% decline in NAD^+^ levels in the heart. Expression of NAD^+^ salvage enzyme NAMPT was decreased and, an increase in NMRK2 enzyme that converts nicotinamide riboside (NR) to nicotinamide mononucleotide (NMN) was noted. This was consistent with results obtained from failing human hearts. NR administration prevented heart failure in mice with dilated cardiomyopathy and partially improved cardiac function in transverse aortic constriction (TAC) mouse model by enhancing NAD^+^ metabolome and cardiac citrate metabolism (30). A genetic model of mitochondrial complex I dysfunction (Ndufs4; cKO) was shown to be more susceptible to heart failure which was accompanied with increased NADH/NAD^+^ ratio and protein hyperacetylation (31). Administration of NMN or overexpressing NAMPT normalized NADH/NAD^+^ ratio and reduced protein acetylation; and thus prevented heart failure in the Ndufs4 cKO and chronic pressure overload models (31).

**Figure 1:**
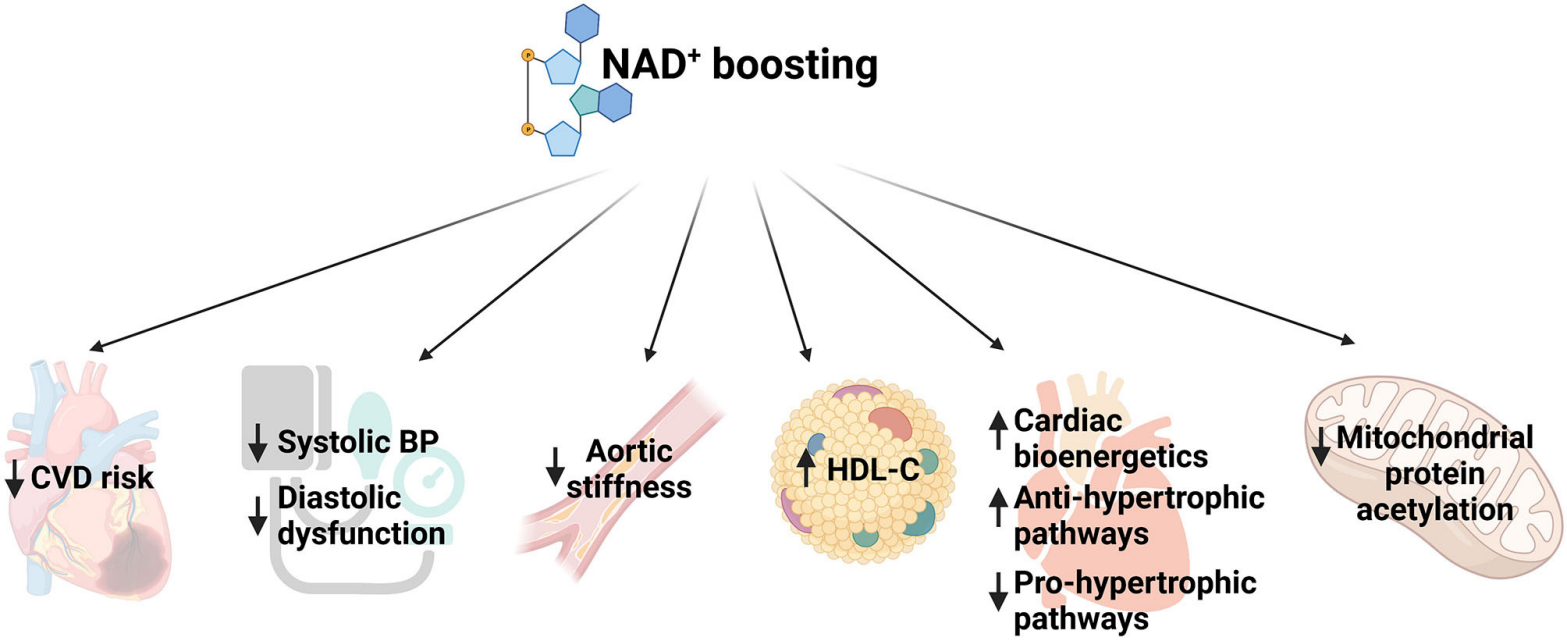
Schematic representing function of NAD^+^ boosting in multiple aspects of cardiovascular diseases. CVD, cardiovascular disease; BP, blood pressure; HDL, high density lipoprotein.

Clinical trials with various NAD^+^ precursors such as Trp, NA, or NR showed that increasing NAD^+^ levels is associated with reduced risk of developing CVD, lowers blood pressure and improves hypocholesteremia, enhances cardiac mitochondrial function and decreases aortic stiffness (32) (Figure 1). For example, in a long term safety and efficacy study, niacin treatment showed lower cholesterol levels and decreased occurrence of non-fatal myocardial re-infarction which led to 11% decrease in mortality compared to placebo (33). Another study showed that elevated plasma tryptophan level is associated with decreased CVD incidence (34). A systematic review reported that niacin treatment increased levels of serum high-density lipoprotein (HDL) levels (35). Moreover, a randomized controlled trial showed that NR administration for 6 weeks led to reduction in blood pressure and aortic stiffness in middle-aged and older adults (36). A recent study reported that diastolic heart failure in humans is associated with depleted NAD^+^ content (37). Using three murine models of heart failure, (i) aging, (ii) hypertension, and (iii) ZSF1 obese rats, the study showed that NAM treatment prevented diastolic dysfunction by improving cardiometabolic function and bioenergetics (Figure 1). Moreover, they performed a long-term population-based analysis which suggested that supplementation of NA or its equivalent is capable of reducing blood pressure and, thereby it was associated with lower risks of cardiac mortality (37).

Since PARP1 hyperactivation has been implicated as a cause of NAD^+^ depletion in various pathologies, pharmacological inhibitors of PARP1 have been tested in pre-clinical models. An *in vitro* study showed that PARP1 inhibition protects tachypacing-induced and gamma-irradiation-induced cardiomyocyte dysfunction in HL1 and rat atrial cardiomyocytes (38). In a mouse TAC model of heart failure, PARP1 inhibition improved cardiac contractile function (39). Moreover, in murine models of chronic heart failure and ischemia/reperfusion (I/R) injury, PARP inhibition has been found to be protective (40, 41). Using a genetic model of mouse preeclampsia, a study reported that deletion of NAD^+^ consuming enzyme CD38 gene leads to reduced blood pressure in this model. CD38 inhibitors are also available which could be tested in CVD models (42). However, such inhibitors may not be highly selective; and as these enzymes are involved in various pathways and tissue functions, their global inhibition may pose unwanted side effects for human application.

## NAD^+^ Precursors and Concerns Associated With Their High Dose Administration

Although several studies have successfully demonstrated the beneficial effects of NAD^+^ boosting, the dosage and precursors used need to be carefully evaluated for human application. Studies conducted have tested Trp, NA, NAM, NAM-mono nucleotide (NMN) and NR as NAD^+^ precursors. As Trp is also involved in other biosynthetic pathways, it seems to be less efficient in elevating NAD^+^ levels in disease conditions (1). NA has lipid lowering effects in blood but at doses higher than 50 mg/day it can cause flushing (43, 44). On the other hand, high doses of NAM have been reported to inhibit SIRT1 (45). NA and NAM have also been shown to affect the cellular methyl pool which is critical for maintaining DNA and protein methylation states. NAM accepts methyl groups from S-adenosylmethionine (SAMe) by the action of nicotinamide N-methyltransferase (NNMT) and generates methylated NAM (MeNAM) and homocysteine. Under physiologic conditions, methylation of NAM is regulated as the K_m_ value of NNMT for NAM is much higher compared to plasma levels of NAM. Thus, high doses of NAM treatment will lead to NNMT activation which may result in depletion of the methyl pool. Consistent with this, NAM has been reported to cause liver toxicity at high doses (46, 47). For example, one study reported that male rats treated with 1 or 4 grams of NAM for 8 weeks had altered hepatic DNA methylation and homocysteine metabolism; along with increased oxidative damage and insulin resistance compared to untreated rats (48). Another research study showed that healthy rats treated with nicotinamide (500 mg/kg) had an increase in the parameters of hepatic and renal toxicity (46). Moreover, NAM treatment in pregnant rats led to decreased placental and fetal DNA methylation which suggests that high doses of NAM treatment may modify epigenetic profile in offspring (49).

Sterile alpha and TIR motif-containing protein 1 (SARM1), an NADase enzyme, when activated causes axonal degeneration. Accumulation of NMN due to loss of NMNAT2, an enzyme that converts NMN to NAD^+^, or an increase in NMN/NAD^+^ ratio, has been shown to activate SARM1 which leads to axonal death. This raises safety concerns for pharmacological use of NMN in humans, although pharmacokinetics study of NMN administration at 100, 250, and 500 mg in humans did not show any deleterious effects after 5 h of intervention (50–52). NMN treatment in mice at a high dose (2,860 mg/kg, twice daily) for 7 days led to elevated alanine aminotransferase indicating liver toxicity. Moreover, NMN treatment at 1,340 mg/day twice daily in beagle dogs showed an increase in body weight; and elevated serum creatinine and uric acid levels indicating compromised kidney function compared to controls (53).

Pharmacokinetics studies and several clinical trials have reported that NR is effective in enhancing NAD^+^ metabolome and that it is orally available and well-tolerated in humans (10, 36, 54, 55). In a randomized double-blinded placebo-controlled trial, unlike NAM treatment, long term NR administration (56 days) at 10, 300 and 1,000 mg/day doses did not affect levels of plasma homocysteine (55). A rodent study showed that rats treated with 300 mg/kg NR for 21 days displayed a non-significant decrease in incremental swimming performance test compared to non-treated rats (56). However, in aged humans, a double-blinded crossover study showed that NR treatment can acutely enhance physical performance (22). Another study showed that NR supplementation at 1 g/day dosage for 21 days seemed to maintain mitochondrial bioenergetics and elevate NAD^+^ metabolome in muscle despite downregulation of mitochondrial pathways. NR supplementation also led to a decrease in age-associated inflammation in these subjects (10). Here, exercise performance or physical activity was not evaluated which would be essential information to determine the outcome of the study accurately.

## Conclusion

Overall, the findings discussed in this article suggest that NAD^+^ boosting is a promising therapeutic strategy for cardiovascular diseases. However, the choice of NAD^+^ precursor, their appropriate dosing and long-term effects in humans need to be critically evaluated and investigated. Long-term effect of these precursors could be tested in animal models at various doses to determine impact on molecular parameters such as DNA methylation, homocysteine metabolism and SARM1 activation. Exercise performance or physical activity should also be evaluated. In non-medicated, metabolically healthy obese individuals, NAD^+^ boosting for 12 weeks did not improve glucose tolerance even though in pre-clinical models of Type 2 diabetes, NAD^+^ precursor supplementation has been shown to be effective. This may suggest that a therapeutic benefit may only be achieved in conditions with metabolic dysfunction and a preventative benefit may require even longer or repeated treatment periods. Thus, such aspects need to be carefully considered for designing clinical trials.

## Author Contributions

FJ and RB designed the concept. FJ drafted and RB edited and finalized the manuscript.

## Conflict of Interest

The authors declare that the research was conducted in the absence of any commercial or financial relationships that could be construed as a potential conflict of interest.

## Publisher's Note

All claims expressed in this article are solely those of the authors and do not necessarily represent those of their affiliated organizations, or those of the publisher, the editors and the reviewers. Any product that may be evaluated in this article, or claim that may be made by its manufacturer, is not guaranteed or endorsed by the publisher.
